# Introduction. Extent, processes and evolutionary impact of interspecific hybridization in animals

**DOI:** 10.1098/rstb.2008.0055

**Published:** 2008-06-05

**Authors:** Klaus Schwenk, Nora Brede, Bruno Streit

**Affiliations:** 1 Department of Ecology and Evolution, J. W. Goethe-University Frankfurt am MainBiology Campus Siesmayerstraße, 60054 Frankfurt am Main, Germany; 2 Aquatic Ecology, EawagÜberlandstrasse 133, 8600 Dübendorf, Switzerland

**Keywords:** animal hybridization, introgression, literature review

## Abstract

Since the time of Charles Darwin, studies of interspecific hybridization have been a major focus for evolutionary biologists. Although this phenomenon has often been viewed as problematic in the fields of ecology, taxonomy and systematics, it has become a primary source of data for studies on speciation and adaptation. Effects from genetic/evolutionary processes, such as recombination and natural selection, usually develop over extended periods of time; however, they are accelerated in cases of hybridization. Interspecific hybrids exhibit novel genomes that are exposed to natural selection, thus providing a key to unravel the ultimate causes of adaptation and speciation. Here we provide firstly a historic perspective of hybridization research, secondly a novel attempt to assess the extent of hybridization among animals and thirdly an overview of the reviews and case studies presented in this theme issue.

## 1. Introduction

**Figure 1:**
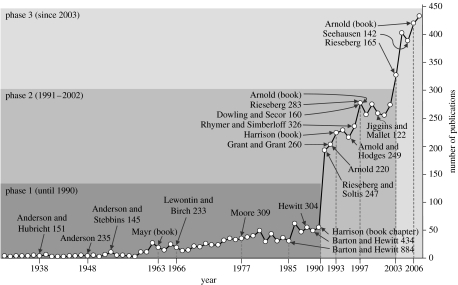
Number of publications on the topic of interspecific hybridization or introgression covering the last 70 years. Data were collected from the ISI literature databank (during December 2007). The selected key publications are highlighted and numbers with the authors indicate the number of citations (Anderson & Hubricht 1938; Anderson 1948; Anderson & Stebbins 1954; Mayr 1963; Lewontin & Birch 1966; Moore 1977; Barton & Hewitt 1985, 1989; Hewitt 1988; Harrison 1990, 1993; Rieseberg & Soltis 1991; Arnold 1992, 1997, 2006; Grant & Grant 1992; Arnold & Hodges 1995; Rhymer & Simberloff 1996; Dowling & Secor 1997; Rieseberg 1997; Jiggins & Mallet 2000; Rieseberg *et al*. 2003; Seehausen 2004).

Research on interspecific hybrids has played a major role in the field of evolutionary biology, since deciphering the mechanisms preventing or allowing interbreeding of species helps to answer general questions such as: How are reproductive barriers between species evolving? Which genes contribute to reproductive isolation? Is gene flow between species (introgression) a creative, additional force in adaptation? During the past 150 years research on interspecific hybridization went through three main phases (figure 1): the first phase is characterized by the search for cases of hybridization and the attempt to develop a theoretical framework; in the second phase many examples of interspecific hybridization were explored using molecular markers; and the third and current phase is fuelled by the rapid integration of genomic and ecological methods. In the first part of this paper we will describe this development from a historical point of view. The second part will then tackle the question of how frequent hybridization occurs in animals, and test the expectation if the level of hybridization varies among taxonomic groups. The last part of the introduction will highlight some of the recent developments in the field illustrated by the contributions to this special issue.

## 2. One hundred and fifty years of hybridization research

Since Darwin first described hybrid formation in the context of speciation, interspecific hybridization has attracted the attention of many evolutionary biologists. Hybrids are formed when different species interbreed, resulting in the combination of genetic material from previously isolated gene pools. Darwin (1859) observed that:

Those forms which possess in some considerable degree the character of species, but which are so closely similar to some other forms, or are so closely linked to them by intermediate gradations, that naturalists do not like to rank them as distinct species, are in several respects the most important to us.

However, until the 1930s the common opinion was that sterility of interspecific hybrids caused the lack of evident hybridization. In the 1930s and 1940s a number of botanists started to experimentally study interspecific hybridization, either using crossing experiments (Anderson & Hubricht 1938) or field studies to estimate the rate of hybridization and to understand the consequences of interbreeding (Anderson 1948). These studies revealed that genetic information was exchanged between species (introgression) and illustrated that introgression was not rare among plants. In the 1960s Mayr (1963) suggested that interspecific hybridization among animals was of minor significance, whereas Lewontin & Birch (1966) indicated that hybridization might represent a source of novel evolutionary trajectories. Their experiments on a fruitfly (genus *Dacus*) suggested that the introduction of genes from another species could serve as the raw material for an adaptive evolutionary advance even though the original hybridization was disadvantageous. During the 1950s and 1960s a number of empirical cases of plant and animal hybridization led to the development of models of hybridization, which were then discussed in the seminal review by Moore (1977). Based on additional empirical evidence generated during the 1970s and early 1980s, Barton & Hewitt (1985) compiled their comprehensive, and very influential, review of instances of interspecific hybridization; they concluded that the majority of cases were explained by a balance between dispersal of parental taxa into a (usually narrow) hybrid zone and selection against hybrids. Another major milestone in the field of interspecific hybridization was presented by Harrison (1990). He concluded that although ample examples of interspecific hybridization had been reported, no single scenario or hybrid model seemed to explain all cases. Instead, the diversity in the origin, dynamics and fate of hybrids suggested that multiple evolutionary pathways were responsible for this phenomenon. Although these landmark reviews fuelled future research, a technological invention, i.e. the polymerase chain reaction, caused a revolution in the studies of hybridization. The number of publications increased fourfold to approximately 200 publications per year (figure 1). During this second phase many studies focused on detailed genetic analyses, hybrid fitness and selection in hybrid zones. The latter issue became a prominent controversy in the field; either selection against hybrids was considered to be independent of environmental factors (tension zone models), or fitness of different genotypes varies in response to environmental variation (e.g. hybrid superiority model) and thus fitness of hybrids is at least equal to that of the parental species. Although historically environmental independent scenarios have been mainly proposed by zoologists and the environmental dependent scenarios preferentially by botanists, this taxonomic division has been softened during the 1990s, and an integrative scenario was presented by Arnold (1997). Owing to the technological advances that allowed the simultaneous measurement of genetic variation at nuclear and mitochondrial loci, the issue of gene flow among species (i.e. introgression) became a major focus in the field of interspecific hybridization of animals (Dowling & Secor 1997). The third phase of hybrid research has been driven by the availability of multiple nuclear markers, i.e. microsatellite DNA, AFLP and SNP, and the first genomic studies of hybrid taxa (Rieseberg *et al*. 2003; Mallet 2005; Rogers & Bernatchez 2007). One of most controversial issues since the 1960s has concerned the adaptive significance of interspecific hybridization. Is hybridization a significant evolutionary mechanism which creates opportunities for adaptation and speciation, or is it just some ‘evolutionary noise’ (Arnold 1997)? Genomic studies of animal and plant systems provided evidence for the first scenario, since hybridization did indeed facilitate major ecological transitions (Rieseberg *et al*. 2003; Gompert *et al*. 2006; Rogers & Bernatchez 2007). These and other intriguing cases of interspecific hybridization have led to the development of new conceptual models on hybridization, which either focus on adaptive radiations or introgression and adaptation (Seehausen 2004; Arnold 2006).

An overview of the past 150 years of hybridization studies illustrates two aspects that are most likely apply to many other fields. Firstly, scientific progress is largely driven by technological advances. Secondly, questions raised 100 years ago are still in the focus of current research projects. The latter is most likely explained by the remaining gap in the recent integration of genetics and ecology. Although we have experienced a fruitful exchange of ideas among both fields of research, the field of molecular ecology has been mainly characterized by the application of selectively neutral markers, such as mitochondrial or microsatellite DNA, to address ecological questions. Mainly, these studies used genetic information to identify species to measure the effect of gene flow or to reconstruct phylogenetic relationships or phylogeographic patterns. However, the genetic basis of natural selection and reproductive isolation were examined only recently, and thus there are still relatively few ecological genetic analyses that have gathered data concerning functional genes, neutral loci and hybrid fitness. Yet, these few studies have illustrated, for example, how introgressive hybridization contributes to the evolution of interbreeding species (Grant & Grant 2008).

We would also like to stress two other, more general, aspects emerging from studies using literature or other databases. On the one hand, they illustrate the additive value of multiple studies of the same phenomenon over a broad taxonomic range, such as pure taxonomic or barcoding studies (Hebert *et al*. 2003). Thus, only the availability of focused, individual studies and structured databases allows us to extract patterns beyond the scope of single observation. On the other hand, our finding that the frequency of hybridizing taxa might represent a neutral by-product of evolutionary change is consistent with similar studies looking at the frequency of cryptic species (Pfenninger & Schwenk 2007). This study also supports the hypothesis that neutral processes are frequently underestimated in evolutionary studies (Lynch 2007).

## 3. Extent of interspecific hybridization among animals

During the recent past, a number of reviews on interspecific hybridization illustrated an unexpectedly wide taxonomic range and, depending on the group of organisms, relatively high frequencies of interbreeding species, e.g. among vascular plants (25%), European butterflies (12%), fruitflies (1%), birds (10%), British ducks (25%) and European mammals (6%; Grant & Grant 1992; Mallet 2005). These figures suggest that interspecific hybridization represents a group-specific process, e.g. ducks have been impacted much more by introgression than have mammals. However, since taxonomic groups are not randomly chosen, but based on available data, they might be biased due to organismic preferences of research teams. Many diverse taxonomic groups may be under-represented in the literature and estimates of the degree to which their species participate in hybridization is thus unknown. Here we present a comprehensive analysis of hybridizing species in the animal kingdom, which includes a correction for study (i.e. organismic) bias. This allows us to test the hypothesis of differential hybridization rates among major metazoan groups. This literature survey is based on a recently developed approach to detect the rate of ‘cryptic species’ in animals (Pfenninger & Schwenk 2007).

### (a) The ‘neutral hypothesis of hybridization’

**Figure 2:**
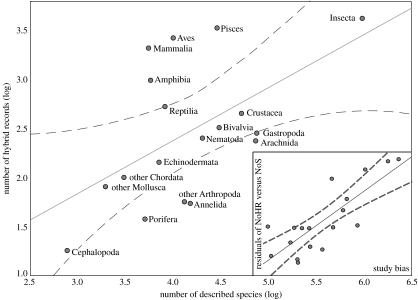
The log of hybrid reports as a function of the log of described species in the respective taxon. Dashed lines represent 95% confidence limits. Residuals of this regression represent either true differences of taxonomic groups or a study bias. In order to test which of the two contributes most to this deviation, we correlated the residuals to the study bias (deviations of a regression of the number of records in the database with the number of the described species). The regression of both residuals resulted in a significant relationship (inset figure).

We have used the publicly available literature database ‘Zoological Record’ (1947–2007). We found 21 972 publications concerning hybrid animals within the field of ecology, evolution and systematics (table 1). In order to test if the abundance of hybrids is equally distributed among various taxonomic groups, we correlated the number of hybrid records with the estimated number of described species (Baillie *et al*. 2004) in each taxonomic group (figure 2). A first significant correlation between both parameters indicates that the level of hybridization is predicted by the number of species per taxon (*R*^2^=0.29, *p*=0.02). The deviations of the regression line (residuals) are composed of differences in study intensity and taxonomic practice in the respective research community, true differences among taxonomic groups and random error. From a second correlation between all records used in our study and the number of described species per taxonomic group, we isolated the residuals as an indicator of a study bias (*R*^2^=0.34, *p*=0.01). Some taxa, especially vertebrates and the economically important pests and crops, have received far greater attention from taxonomists and population geneticists than most other taxa (e.g. marine invertebrates). This parameter explained a large part of the residual variation derived from the first regression (*R*^2^=0.66, *p*=0.00004). This pattern indicates that the rate of interspecific hybridization is relatively homogeneous among animal groups and only a few groups deviate from this pattern. Thus, the level of interspecific hybridization might represent an inherent and neutral parameter of evolutionary change. This analysis does not reveal anything about the potential significance of hybridization, or the frequency of backcrossing and introgression. However, it implies that in each taxonomic group we have *a priori* similar chances for gene exchange among species. We do not know how accurately the hybrid records reflect the actual number of interspecific hybrids. However, if the proportion of multiple hits in the number of hybrid records and the total number of records is roughly the same, we estimate the average hybridization rate to be approximately 1% (range 0.1–3%). This relatively low frequency of hybridization in animals is certainly at odds with previous estimates, which have predicted a frequency of approximately 10% (Mallet 2005). This discrepancy raises the question whether previous studies overestimated rates of hybridization due to taxon bias and taxonomic practice, or if a literature survey underestimates the rate since non-hybridizing taxa might on average be more frequently studied than hybridizing taxa. Future studies that focus on other kingdoms, such as plants and fungi, might allow us to better understand the potential impact of interbreeding.

But even if the hybridization frequency is low, we would argue that the studies of interspecific hybridization should remain a major focus of evolutionary biology for two reasons. Firstly, although the frequency of individuals which actually interbreed with other species might be rare, the occurrence of occasional hybridization events might be sufficient to cause long-lasting changes in the genomic architecture of species. Thus hybridization might be rare but if it occurs it has a major impact on the origin and fate of evolutionary lineages. Secondly, hybridization provides a natural setting to study efficiently the interaction between genetic and ecological differentiation. In an attempt to justify the study of rare phenomena, Vrijenhoek (1989) once argued: ‘Examination of unusual processes opens a window to understanding what is common and what is normal. We learn about human health by studying innumerable disorders and diseases, and we learn about the function of normal genes by studying the phenotypic consequences of mutations. To understand the functional properties of outcrossing, biparental sexuality, we must study the consequences of aberrant reproductive processes.’ This argument applies to studies of ‘rare’ hybridization as well.

## 4. The process and its evolutionary impact

By combining previously isolated gene pools, interspecific hybridization results in the origin of new genotypes. If this phenomenon is viewed at the level of populations, then interspecific hybridization can also result in significant shifts of allele frequencies. When compared with mutation and recombination within species (the more usual mechanisms for the origin of new genotypes), interspecific hybridization can result in rapid and long-lasting changes among interbreeding species. The evolutionary change due to hybridization can occur within one generation, thereby exposing new gene combinations to natural selection. Furthermore, by compressing the time scale over which evolutionary processes occur, and by allowing the formation of new gene combinations, interspecific hybridization enables investigations into the processes of natural selection. Owing to these properties, examples of interspecific hybridization have been described as ‘natural laboratories for evolutionary studies’ (Hewitt 1988), ‘windows on evolutionary process’ (Harrison 1990) or as ‘an ecologically dependent behavioural phenomenon, with genetic consequences’ (Grant & Grant 2008). In the following subsections, we will address several topics of current hybridization research exemplified by the contributions to this theme issue.

### (a) Introgressive hybridization

During the past decades, introgressive hybridization became one of the well-studied phenomena in evolutionary biology. This development is explained by two achievements. Firstly, advances in the field of genomics have allowed a definition of introgression at an unprecedented level of detail (Arnold 2006). Secondly, there has been a simultaneous series of analyses that have addressed the possible evolutionary impact of introgression events. For example, combined analyses of mitochondrial and multiple nuclear markers have defined the parental contributions to hybrid genomes. Studies based on these technological advances supported two main conclusions: (i) introgression is much more frequent than previously thought and (ii) hybridization can play an important creative role in adaptive evolution (Arnold *et al*. 2008). The unambiguous detection of introgression is not trivial since processes such as lineage sorting (i.e. retention of ancestral polymorphisms) can result in identical patterns of genetic differentiation (Alves *et al*. 2008). However, it is even more difficult to determine the evolutionary importance of introgression among species (Futuyma & Shapiro 1995). Yet, comprehensive studies on the Darwin's finches (*Geospiza*) have revealed, for example, the effect of introgressive hybridization on developmental genetic pathways of phenotypic traits (i.e. beak shape; Grant & Grant 2008).

### (b) Identification of hybrid classes

Owing to the application of numerous genetic techniques and the recent availability of advanced analytical tools, identification of interspecific hybrids as well as parental and backcross classes have become a relatively straightforward exercise. In particular, software based on Bayesian inference such as Structure and NewHybrids provides a powerful discriminatory tool (Excoffier & Heckel 2006; Vaha & Primmer 2006). In this theme issue, Anderson (2008) presents an extension of the frequently used software NewHybrids for the detection of hybrid classes. In addition, the application of various molecular approaches for the identification and analysis of hybridization is presented by Vigfúsdóttir *et al*. (2008).

### (c) Prezygotic reproductive isolation

If interspecific hybridization is costly, then selection might favour the evolution of prezygotic isolating mechanisms (e.g. mating behaviours) that reduce heterospecific matings. This process will subsequently enhance reproductive isolation between species and result in reinforcement. If hybridization is less costly, or provides fitness advantages, prezygotic isolation might be weak and allow hybridization events. Even though these events might occur at a low frequency they are bound to have long-lasting consequences for the genetic architecture of populations due to gene flow among species. How behaviour of males and females determines the outcome of hybridization is illustrated in the studies of den Hartog *et al*. (2008), van der Sluijs *et al*. (2008) and Stelkens *et al*. (2008).

### (d) Mating biology

Many studies on interspecific hybridization have focused on aspects of mating biology, since parental taxa and interspecific hybrids are often characterized by significant shifts in reproductive modes (i.e. from sexual to asexual) and ploidy levels (Schultz 1969). In this context, interspecific hybridization provides a natural setting to study the origin and genetic background of major changes in mating biology. A change in the mode of reproduction might facilitate (i) the geographical expansion of interspecific hybrids (Choleva *et al*. 2008), (ii) the origin of new lineages (Lampert & Schartel 2008), (iii) unexpected patterns in social insects (Feldhaar *et al*. 2008), or (iv) speciation (Cunha *et al*. 2008).

### (e) Ecological differentiation and conservation genetics

One of the main topics in the field of interspecific hybridization is related to the question of whether the selection against hybrids is environmentally independent or dependent (i.e. reflecting endogenous or exogenous selection, respectively). The spatio-temporal distribution of plankton species and hybrids along environmental gradients suggests the action of exogenous selection (Petrusek *et al*. 2008), and the geographical distribution of species and hybrids indicating a role for man-made habitat disturbances, such as lake eutrophication (Keller *et al*. 2008). Anthropogenic disturbances, including the invasion of species or biomanipulation, not only have an impact on biodiversity and the ecology of systems but also on the evolutionary fate of organisms. The introgression between wild and feral domestic cats is an example of the effects that anthropomorphic habitat modifications may have (Oliveira *et al*. 2008).

## 5. Synopsis

Futuyma & Shapiro (1995) mentioned in their review of the 1993 book edited by Harrison that ‘Arbitrarily chosen molecular and morphological markers have provided abundant insight into hybrid zones, but they have not answered some of the most difficult and important questions: on what genes and characters does selection act, and what are the agents of selection? …most of the work lies ahead of us.’ Since the 1990s, we have witnessed a tremendous increase in the number of studies on interspecific hybridization, but only during the last few years has it been feasible to study simultaneously multiple functional genes and selectively neutral markers. Presently, we have the tools to examine instances of interspecific hybridization in unprecedented detail. In particular, the analyses of neutral markers and candidate genes, in combination with life-history experiments, have the potential to unravel central questions in evolutionary biology. In addition, a number of currently controversial issues in biology are related to the field of interspecific hybridization, which are as follows. (i) What are the consequences of hybridization among genetically modified organisms and wild congeners? (ii) What are the consequences of interbreeding between invasive and indigenous species? (iii) How will global climate changes contribute to species invasions and hybridization? (iv) What is the role of genetic exchange in the evolution of organisms? (v) To what extent does hybridization determine the fate of endangered species?

Although previous studies identified certain taxonomic groups that are known to have accelerated levels of interspecific hybridization, our literature survey suggests that hybridization represents a phenomenon distributed uniformly across the animal kingdom. As the historical overview, the reviews and the case studies presented in this issue demonstrate, interspecific hybridization remains a growing and exciting field in evolutionary biology.

## Figures and Tables

**Table 1:** Results of database search in Zoological Record (1947–2007) for several metazoan taxa. (Information on the estimated number of currently described species for each taxon was obtained from the IUCN 2004 report; Baillie *et al*. 2004.)

	number of		
	
taxa	hybrid records	total records	described species
Arthropoda			
Insecta	4291	427 279	950 000
Crustacea	452	91 275	52 000
Arachnida	242	58 445	73 000
other Arthropoda	57	19 361	13 000
Chordata			
Mammalia	2094	180 409	5416
‘Amphibia’	999	33 864	5743
‘Reptilia’	536	58 526	8163
Aves	2685	186 024	9917
‘Pisces’	3393	161 095	28 500
other Chordata	100	4715	3025
Mollusca			
Cephalopoda	18	10 952	768
Bivalvia	323	25 320	30 000
Gastropoda	289	34 557	75 000
other Mollusca	81	39 950	1950
Annelida	55	20 536	15 000
Echinodermata	144	16 522	7000
Porifera	38	6384	5000
Nematoda	253	31 866	20 000
